# A Rare Case of High-Dose Methotrexate Toxicity Precipitated by Piperacillin-Tazobactam: Delayed Clearance, Acute Kidney Injury, and Rescue with Glucarpidase

**DOI:** 10.7759/cureus.103715

**Published:** 2026-02-16

**Authors:** Hashir Abdullah, Hassan Waraich, Kainat Atta, Fatima Atta

**Affiliations:** 1 Internal Medicine, Manchester University NHS Foundation Trust, Manchester, GBR; 2 Ear, Nose, and Throat, Countess of Chester Hospital NHS Foundation Trust, Manchester, GBR; 3 Accident and Emergency, Royal Berkshire Hospital, Reading, GBR

**Keywords:** acute kidney injury, cns lymphoma, high grade lymphoma, methotrexate, methotrexate neurotoxicity, methotrexate toxicity, piperacillin-tazobactam, tazocin

## Abstract

One of the fundamental pillars of central nervous system (CNS)-directed therapy of aggressive lymphomas is high-dose methotrexate (HD-MTX). The use of this drug is offset by a well-defined risk of renal pharmacokinetic retardation and extreme toxicity, often compounded by drug-drug interactions, especially penicillin-class antibiotics. A 58-year-old female patient who had primary high-grade B-cell lymphoma was administered high-dose methotrexate (HD-MTX) standard prophylaxis. Piperacillin-tazobactam (PTZ) was empirically initiated within 24 hours, which is against documented avoidance instructions. The 24-hour MTX level was critically high at 193 umol/L, and acute kidney injury (creatinine increase: 74 to 259 umol/L) was also present. The administration of glucarpidase was performed approximately 48 hours after the administration of MTX, after folinic acid escalation and consultation with toxicology. Renal performance improved without dialysis, and the MTX levels dropped. The importance of the clinically significant interaction of HD-MTX and PTZ is critically highlighted in this case. It brings out the urgency of rigorous antimicrobial stewardship, the life-saving significance of timely glucarpidase, and reveals the system-level crises in medication visibility and electronic prescribing safeguards.

## Introduction

High-dose methotrexate (HD-MTX), which is doses that are greater than 1 g/m², is a vital part of curative regimens in hematological malignancies, specifically for central nervous system (CNS) prophylaxis and treatment [1]. Its efficacy depends on rapid renal clearance, which can be blocked by the comorbid effects of nephrotoxins or by agents that competitively inhibit renal tubular secretion. Slow clearance causes systemic exposure, which may cause potentially fatal complications such as severe mucositis, myelosuppression, and worsening nephrotoxicity [2].

Another interaction that is rather dangerous but well-documented is between MTX and penicillin analogs, including piperacillin [3,4]. The agents compete with one another in terms of active secretion through renal organic anion transporters (OAT1/OAT3), directly affecting MTX secretion and increasing plasma concentrations. Although acknowledged, it is still a common source of avoidable HD-MTX toxicity. Glucarpidase (carboxypeptidase G2) is an essential rescue agent, which degrades extracellular MTX to inactive metabolites and therefore offers an extra-renal route of elimination for patients with acute kidney injury (AKI) and markedly elevated MTX levels [5].

This report provides a paradigmatic instance of HD-MTX toxicity caused by piperacillin-tazobactam (PTZ). The case has great learning potential, as it shows how quickly and intensely this particular drug interaction develops and demonstrates how glucarpidase works. It also includes a root-cause analysis that exposes gaps in the electronic health record (EHR) integration and prescribing safety.

## Case presentation

Patient history & background

A 58-year-old woman with a complicated oncology history, including a case of primary high-grade B-cell lymphoma (on the right breast) and hormone receptor-positive breast carcinoma, was to receive CNS prophylaxis with HD-MTX. She had undergone six cycles of Rituximab, Cyclophosphamide, dHydroxydaunorubicin (doxorubicin), Oncovin (vincristine), and Prednisone (R-CHOP regimen) for her lymphoma. Her medical history included morbid obesity (BMI 42 kg/m^2^), fibromyalgia, and a history of a line-related deep vein thrombosis. The baseline renal functioning was normal (serum creatinine 74 μmol/L, estimated glomerular filtration rate (eGFR) exceeding 90 mL/min/1.73m^2^).

Pre-treatment protocol & documentation

The patient was hospitalized on day one and admitted voluntarily. The chemotherapy protocol involved forced intravenous hydration, alkalinization of the urine (urine pH above 7.5), and leucovorin rescue. The severe written protocol included an explicit prohibition on non-steroidal anti-inflammatory drugs (NSAIDs), co-trimoxazole, and penicillin-based antibiotics until the serum concentration of MTX was assured to be less than 0.1 μmol/L [1-3]. This ban was recorded on the physical chemotherapy prescription chart but was not incorporated into the electronic prescribing (e-prescribing) alert system at the hospital.

On day three, after confirming adequate urinary alkalinization (pH 8.5), HD-MTX (4 g/m^2^) was administered. The actual infusion times used for starting and stopping the infusion were not documented in the electronic health record (EHR).

About 12 hours after MTX infusion (on the evening of day three), the patient experienced vomiting and reported a subjective fever. The on-call resident doctor without immediate access to the physical chemotherapy record empirically prescribed PTZ (4.5g IV q8h) via the e-prescribing system.

On the morning of day four (i.e., approximately 24 hours post-MTX), the patient's routine blood tests showed a sharp increase in serum creatinine to 194 μmol/L. The timed 24-hour post-MTX level returned with a value of 12 μmol/L, an extremely toxic level. PTZ was immediately stopped and replaced with meropenem.

Diagnostic investigations & specialist management

Serial biochemistry confirmed AKI (creatinine peak ~250μol/L) in direct temporal association with PTZ exposure (Tables 1, 2).

**Table 1:** Electronic patient records during the acute toxicity phase Selected lab results show the simultaneous increase of serum creatinine and the reported toxic level of methotrexate (MTX). All samples taken on the same day (Jul 5) at various times - Sample 1 at 22:38; Sample 2 at 18:57; Sample 3 at 16:03; Sample 4 at 04:34. AKI: Acute Kidney Injury; eGFR: estimated glomerular filtration rate; ALT: Alanine aminotransferase; H: High; L: Low.

Parameter	Sample 1	Sample 2	MTX level 1	Sample 3	MTX level 2	Sample 4	Reference range	Units
Sodium	141	140	-	139	-	143	133-146	mmol/L
Potassium	4.3	4.2	-	4.3	-	4.4	3.5-5.3	mmol/L
Creatinine	259 (H)	255 (H)	-	243 (H)	-	194 (H)	49-90	μmol/L
Urea	6.9	6.6	-	6.3	-	5.1	2.5-7.8	mmol/L
AKI status	... A	... A	-	... A	-	... A	-	-
eGFR (estimated)	15 (L)	16 (L)	-	16 (L)	-	21 (L)	>60	mL/min/1.73m^2^
Albumin	21 (L)	24 (L)	-	22 (L)	-	23 (L)	35-50	g/L
Bilirubin	12	17	-	17	-	17	<21	μmol/L
Alkaline phosphatase	54	59	-	55	-	51	30-130	μ/L
ALT	15	16	-	16	-	16	<=55	μ/L
Calcium	1.84	1.85	-	1.83	-	-	-	mmol/L
Adjusted calcium	2.13 (L)	2.11 (L)	-	2.11 (L)	-	-	2.20-2.60	mmol/L
Phosphate	1.70 (H)	1.57 (H)	-	1.52 (H)	-	-	0.80-1.50	mmol/L
CRP	-	-	-	134.3 (H)	-	64.7 (H)	<=5.0	mg/L
Methotrexate	-	-	77.56	-	193.00	-	-	μmol/L

**Table 2:** Electronic patient records during the recovery phase Selected laboratory results indicate that the renal functioning has stabilized, and the methotrexate (MTX) levels are decreasing. Sample 1 (Jul 25) at 04:42; Sample 2 (Jul 24) at 05:38; Sample 3 (Jul 23) at 04:48; Sample 4 (Jul 22) at 07:37. AKI: Acute Kidney Injury; eGFR: estimated glomerular filtration rate; ALT: Alanine aminotransferase; H: High; L: Low.

Parameter	Sample 1	Sample 2	MTX level 1	Sample 3	MTX level 2	Sample 4	Reference range	Units
Sodium	139	141	-	143	-	146	133-146	mmol/L
Potassium	3.3 (L)	3.4 (L)	-	3.8	-	3.5	3.5-5.3	mmol/L
Creatinine	122 (H)	134 (H)	-	143 (H)	-	163 (H)	49-90	μmol/L
Urea	5.8	6.9	-	7.9 (H)	-	11.2 (H)	2.5-7.8	mmol/L
AKI status	...	...	-	...	-	... A	-	-
eGFR (estimated)	37 (L)	33 (L)	-	31 (L)	-	26 (L)	>60	mL/min/1.73m^2^
Albumin	28 (L)	28 (L)	-	24 (L)	-	25 (L)	35-50	g/L
Bilirubin	10	9	-	8	-	7	<21	umol/L
Alkaline phosphatase	111	132 (H)	-	132 (H)	-	149 (H)	30-130	μ/L
ALT	19	20	-	18	-	18	<=55	μ/L
Calcium	-	2.15	-	2.08	-	2.08	-	mmol/L
Adjusted calcium	-	2.35	-	2.34	-	2.32	2.20-2.60	mmol/L
Magnesium	-	0.73	-	0.68 (L)	-	0.73	0.70-1.00	mmol/L
Phosphate	-	0.82	-	0.84	-	1.13	0.80-1.50	mmol/L
CRP	8.8 (H)	12.0 (H)	-	14.1 (H)	-	20.3 (H)	<=5.0	mg/L
Methotrexate	-	-	0.08	0.11	0.09	0.11	-	μmol/L

Further information about methotrexate toxicity was obtained from Toxbase (www.toxbase.org). Given the toxic level of MTX and the changing renal function, urgent glucarpidase administration was suggested [4,5]. Per protocol, leucovorin was not given for two hours before glucarpidase dosing [5].

This included the following treatments: early morning on day five (~48 hours post-MTX start), Glucarpidase (Voraxaze(R)), 5550 units (approximately 50 units/kg) as one intravenous bolus was administered to the patient.

Outcome & follow-up

Following glucarpidase administration, the patient's renal function stabilized (Table 3) and renal replacement therapy was unnecessary.

**Table 3:** Timeline of key events HD-MTX: high dose methotrexate; PICC: Peripherally inserted central catheter; PTZ: piperacillin-tazobactam; MTX: Methotrexate; AKI: Acute Kidney Injury; eGFR: estimated glomerular filtration rate.

Date/Time	Event
02/07/2023	Elective admission for first HD-MTX; written directive to avoid penicillin/NSAIDs/co-trimoxazole until MTX
03/07/2023	PICC insertion; hydration and alkalinisation ongoing.
04/07/2023	Pre-chemo checks: urine pH 8.5; HD-MTX commenced (exact infusion time not recorded).
04/07/2023 evening	Vomiting; reported fever; PTZ started (21:00 q8h)
05/07/2023	Creatinine 194 µmol/L; PTZ stopped; meropenem started.
05/07/2023	24-hour MTX 12 µmol/L; toxicology engaged; glucarpidase arranged.
06/07/2023	Glucarpidase 5,550 units IV administered; folinic acid withheld 2 h pre-dose.
07/07/2023 onward	AKI stabilised (~259 µmol/L); close monitoring; renal referral; MTX followed to near <0.1 µmol/L.

Serial MTX levels were monitored keeping in mind that standard immunoassays show cross-reactivity with glucarpidase metabolite (DAMPA) for about 48 hours, and could therefore provide falsely elevated readings [5]. The patient recovered and was able to go on with subsequent planned oncological management.

## Discussion

The case is a classic example of a preventable, iatrogenic interaction of drugs, which resulted in severe harm to patients and the use of resources [1,2].

Pathophysiological mechanism and causal attribution

This chronology is diagnostically definitive: the establishment of PTZ during the hours after infusion of HD-MTX was preceded by a rapid increase in serum creatinine and a toxic concentration of MTX in 24 hours. This is in line with the competitive inhibition of renal tubular secretion through shared organic anion transporters (OAT1/OAT3) [3,4]. Although inter-individual pharmacogenomic variation may play a role in affecting kinetics, the immediate and dramatic impact in this case is due to the drug interaction.

Rescue pharmacology: reason and reasonableness

The presence of standard rescue is a base, but not enough in the case of developed AKI and extremely high levels of MTX. Glucarpidase is one of the definitive interventions [5]. It is advised that it is most effective when used in the 48-60 hour post-infusion of HD-MTX. The case-reported management, namely a quick toxicology consultation, compliance with the leucovorin hold period and the timely administration is the best-of-the-best. The interpretive pitfall of MTX immunoassays is one of the critical post-administration factors that must be taken into account because it cross-reacts with DAMPA and may lead to creating a false impression of updated active MTX levels within 48 hours.

Imperatives of systems analysis and quality improvement

This event shows there are serious systems flaws:

Information Silos

The ban against all forms of penicillins, which were life-threatening, was given only in the physical record that was inaccessible to the e-prescribing clinician.** **

Poor Decision Support

The e-prescribing system did not have a hard-stop or high-severity interaction notification of penicillin-class antibiotics in patients having an active HD-MTX order. 

Documentation Lapses

The inability to note the actual time of MTX infusion initiation compromises the accuracy of the interpretation of timed levels and rescue levels. 

Antimicrobial Stewardship in Specialties

This case endorses the complete contraindication of penicillin derivatives during the HD-MTX clearance window.

## Conclusions

This report is an example of how a familiar pharmacokinetic interaction between a combination of PTZ and HD-MTX can trigger an acute renal injury within a few hours and a fatal decrease in excretion of the drug. The achievement of success depended on the conscious awareness and the prompt application of glucarpidase. To avoid the same, healthcare facilities should implement strong technological protection systems, such as built-in EHR alerts as well as hard-stop prescribing to make chemotherapy-related contraindications apparent at the point of care across all facilities.
